# Legal Logit Model for predicting judicial disagreement in Indian courts

**DOI:** 10.3389/frai.2025.1671474

**Published:** 2025-10-22

**Authors:** Sivaranjani N, Jayabharathy J

**Affiliations:** ^1^School of Computer Science and Engineering (SCOPE), Vellore Institute of Technology (VIT) Chennai Campus, Chennai, Tamil Nadu, India; ^2^Department of Computer Science and Engineering, Puducherry Technological University, Puducherry, India

**Keywords:** difference of opinions, affirmative and reverse decisions, choice modeling, conditional logit, logit model, SDG 17

## Abstract

Once a case reaches the Supreme Court on appeal, the justices may either affirm or reverse the judgment of the lower court. Forecasting such judicial disagreement is important not only for predicting outcomes but also for understanding the judge-specific and case-specific factors that drive these decisions. This study aimed to present the Legal Logit Model (LLM), an evolved neural network-based version of the Multinomial Logit (MNL) model. The LLM combines the interpretability of discrete choice theory with the flexibility of neural networks. Therefore, it is capable of modeling complex, non-linear interactions while preserving transparency about the influence of individual features. Utilizing features extracted from both cases and judges, the model predicts whether the Supreme Court will reverse a lower court's ruling and highlights the factors most strongly associated with disagreement. When tested on a dataset of Supreme Court opinions, the LLM achieves 80% accuracy in predicting outcomes, outperforming conventional logit and deep learning-based models. Despite the possibility of motivated reasoning in Supreme Court opinions, limiting causal interpretation, the findings show that the LLM presents an interpretable and effective predictive framework applicable to the study of judicial decision-making.

## 1 Introduction

Examination of human dynamics has revealed that individuals tend to have a strong inclination to maintain the status quo when making choices among alternatives. The legal domain is one such domain where judges make predictions based on political and socio-legal situations. Answering one or more questions is the starting point of prediction. Should I take a case close by? Regardless of whether to settle the case outside or take it to court? Will the settlement sum be awesome? What are the odds of winning the case? These are a portion of the inquiries that involve predicting the outcome of a case, and legal professionals need to manage them consistently (Sivaranjani et al., 2019). These inquiries address the significance of result expectations in the event of choice, making settlement decisions, and different parts of legal processes (Sivaranjani et al., 2019). Lawful professionals have been investigating the cases reflectively to recognize and comprehend the elements or factors that play a significant part in making decisions. In any case, analyzing decisions after they are made is not the sole strategy for understanding the decision-making process (Surden, 2014), and the logical speculations should be tested against future results. Which factor influences the prediction of the constitutional court decision-making process: legal background or political background? Legal scholars and social researchers have long investigated legal choices to understand what motivates judges. For the past 50 years, research has been using past legal data to describe and predict the court decision-making process. However, existing work to forecast court decision-making has significant constraints. (i) Almost all the studies related to legal focus on the U.S. Supreme Court cases. The U.S. custom-based law framework is guided by the standard of stare decisis, under which previous cases in a given space of the law have precedential impact on future cases. This uniformity between the facts and output enables machine learning to be used in prediction. Additionally, existing work considers the voting behavior of judges in prior cases to obtain the outcome for new cases. Unluckily, the Supreme Court of India lacks this rich source of information due to the unavailability of judges' votes. (ii) Second, none of the current examinations have unambiguously tried to examine the overall commitment of legal perspective vs. political perspective factors to figure out court choices. There is a long-standing discussion about which aspect impacts a judge's decision-making process and subsequently helps to forecast the case. Conventional legal researchers are likely to extract the important legal features and inquire about legal policies as the case arises. Different researchers also focus on non-legitimate elements, such as judges' perspectives or general assessment, for court decision-making. Determining legal results cannot end this adapted discussion by giving causal proof to one side or the other. However, it helps to test the aspect that influences the judge's prediction. The legal scholars argued that the legal perspective alone influences the decision-making, whereas the legal scientist argues that the non-legitimate elements may also influence the decision-making. Hence, this study discusses various aspects that are considered to be influencing factors for a judge to make a judgment. Thus, any judgment given by the court not only considers case-specific features and prior cases but also judges specific features. Out of 8k appeal cases in the Supreme Court, only 928 cases have a difference of opinion between the lower court and the Supreme Court. Though many studies try to predict the behavior of the Supreme Court using prior cases, none of the research has focused on identifying the influencing aspect for any decision. This study examines the factors that influenced a judge to overturn a lower court decision, focusing specifically on the features that influenced the judge's decision in each case. There is a connection between a judge's values and the public policies they uphold, and vice versa: “Judicial judgments are influenced by the judge's perspective of public policy” (Xiao et al., 2018). In particular, certain judges are inclined to arrive at specific verdicts due to social, financial, and cultural shifts, as well as the judge's distinctive character, personal instincts, and lifelong experiences (Luo et al., 2017). The judges may be influenced by (1) direct influences (legal and political experiences, intelligent and emotionally unstable traits) and (2) indirect and remote influences (legal and general education, wealth, and social status). The proposed LLM (Legal Logit Model) takes court opinion as input and suggests how people's preferences vary depending on past experiences and what seems best in the given circumstances. The model is trained to accurately forecast the substantial considerations involved in making a decision.

The study is organized as follows: Section 2 discusses related works and their shortcomings, and Section 3 presents the research motivation. Section 4 explains the dataset collection and analysis. Section 5 introduces the baseline Discrete Choice Model and the proposed Neural Network–based Discrete Choice Model. Section 6 evaluates the proposed model against other models using various performance measures. Finally, Section 7 summarizes the findings, followed by the conclusion.

## 2 Existing work

With the rise of Artificial Intelligence (AI) over the past decade, quantitative legal prediction, which focuses on the ex-ante expectation of future legal outcomes, has become an integral part of analyzing legal issues. Machine learning is nothing but a program that learns from experience, predicts the future, and improves performance (Surden, 2014). The primary reason for AI is to identify examples and connections in information and to infer expectations about future results. Interestingly, conventional causal surmising approaches that make the best-case scenario, hypothesis-driven expectations about future results, and quantitative legal expectations center completely around the anticipated endeavor. Not the illustrative, yet the prescient force of a variable is significant here. Conversely, put in an unexpected way (Katz, 2013). Though many had an idea to predict the constitutional court decisions, only a few models were developed for forecasting the judicial decisions (Holmes, 1897). The first model for legal prediction was developed in 2004 for use in analyzing US Supreme Court decisions. Ruger et al. (2004) developed a model to predict the voting behavior of individual judges and appeal case final decisions. They have also compared the performance of the machine learning algorithm and the human legal experts. This model depends on the observable case features that are extracted directly from the case database. This work is extended by Guimera and Sales-Pardo (2011) to predict the behavior of a judge by considering not only his prior voting behavior but also the voting behavior of 8 other judges in the same case. Another model developed by Katz et al. (2017) is fully predictive. Exclusive feature engineering improves the performance of the model. The features are given to the Random Forest algorithm to predict the court decision. The model (Mackaay and Robillard, 1974) utilizes the nearest neighbor in 64 tax cases using 46 descriptors. The model is developed using the features that have the same values. This work is later extended by SHYSTER (Popple, 1996), which assigns weight to each of the descriptors and uses more complex feature engineering. The model using regression analysis developed by Haar et al. (1977) reduced the number of features from 67 to 32, which were then given as input to the regression analysis. Dependent variables are considered the outcome. Using five case descriptors, Zeleznikow and Hunter (1994) used a decision tree classifier for predicting debt deferral cases. CATO (Aleven, 2003), designed by Aleven et al., extracts similar cases by identifying the factors applicable to the case and uses the similar to predict the case outcome. IBP, developed by Brüninghaus and Ashley (2003), recognizes the issue in the case and the winner of the case. This is then used to predict the out-of-sample trade secret law cases. They have enhanced the model (Ashley and Brüninghaus, 2009) called SMILE+IBP to reason the case text. The model actually classified the text by extracting the facts and forecasted the case outcome using extracted factors. VJAP (Grabmair, 2017), developed by Grabmair, predicted the outcome of trade secret cases and defended the prediction using legal arguments. They have compared the results with the IBP model. Christensen (2018) used logistic regression to predict the behavior of the U.S. Supreme Court in Indian Law cases.

Beyond computer science methodologies, a considerable volume of scholarship across economics, psychology, political science, and empirical legal studies has explored the process of judicial decision-making. For example, political economists such as Ash and Chen (2018) analyze voting decisions by judges using political economy models, while political scientists (e.g., Segal and Spaeth, 1993) privilege ideological and institutional explanations through the framework of the attitudinal model. Moreover, psychological analyses highlight the effect of cognitive bias and heuristic processing among judges (Guthrie et al., 2007). Finally, empirical legal scholarship examines the relative influence of legal and extralegal factors in determining case outcomes. Entering into this literature situates our particular inquiry within a broad interdisciplinary enterprise to understand comprehensively what influences adjudicative behavior.

## 3 Motivation

From the literature review, it is clear that all the work focuses on predicting the decision of the new case based on past similar cases. Some models were developed using machine learning algorithms, while others employed hypothesis testing. All the models focus on increasing the accuracy of the prediction, giving the least importance to feature extraction and factors influencing the judgment. Thus, there was a need to provide a solution for identifying the factor that influenced the judge to make a particular judgment. Furthermore, during the feature selection, some models leave out some features that are related to judgment, but decision-making does not depend only on case-specific features. Thus, research on legal prediction requires all of the attributes to be considered. This motivated the use of the choice model in legal prediction technology, where it not only improves the prediction but also helps to analyze the predictive factors for that decision.

A prediction model is developed by considering the drawbacks of the existing system, called LLM. The extracted features are categorized according to their nature, including judge-specific and case-specific features. The model starts by predicting the outcome of a particular judge in a specific case. The model then uses correlation to learn the influencing parameter [it may be either case-specific features, citation, or the law involved (section code)] that made the judge take a particular decision. In this research, the proposed LLM offers better predictability and, when combined with IIA, provides a deeper understanding of the decision made.

## 4 Dataset

The proposed model relies on the Supreme Court of India dataset, which contains more than ~13 thousand cases with judgments from 1950 to the present. The database is taken from the website http://www.liiofindia.org/in/cases/cen/INSC (Sivaranjani and Jayabharathy, 2022a). Out of ~13k decided cases, there are around eight thousand appeal cases filed in the Supreme Court due to an unsatisfactory decision of the lower court. The judgment given by the Supreme Court has a particular structure on different aspects of the case. Judgments are usually divided into different aspects that cover the entire content of a particular case to allow the standardization of text. The different aspects of the case text (Sivaranjani et al., 2023) are (i) Facts: this subsection contains the issues raised and the factual background of the case; (ii) Law: contains the section codes applicable to case; (iii) Lower Court Decision: this contains the decision given by the lower court; (iv) Case Citation: contains the case decision that impacts the Supreme Court decision; (v) Reasoning: points highlighted by the advocates about the case. This study analyzes which of the aspects influenced the judge to change the decision given by the Supreme Court in appeal cases. Our previous work (Sivaranjani et al., 2021) already classified the new case from the appeal case and predicted the outcome of the appeal case (Sivaranjani and Jayabharathy, 2022b). The appeal case is already decided in the lower court in favor of one of the parties, either the petitioner or the defendant. The party with unsatisfied judgment from the lower court goes for an appeal in the Supreme Court. If the Supreme Court gives the judgment in favor of the petitioner (one who appealed the case), then a difference of opinion arises. Not all the appeal cases will have a difference of opinion.

Limitations of the Dataset: A limitation of our research is that the data set is derived from Supreme Court opinions, which are framed after the final decision. Therefore, information inherent to these opinions might reveal motivated reasoning such that judges feature particular facts and legal arguments supporting their chosen conclusions. Thus, even though the Legal Logit Model (LLM) identifies factors associated with judicial disagreement, such patterns might reveal how outcomes are justified rather than an indication of what highlights factors associated with judicial reversals. Thus, we interpret our results to be predictive correlations but not causal influences upon judicial behavior.

**Figure 1:**
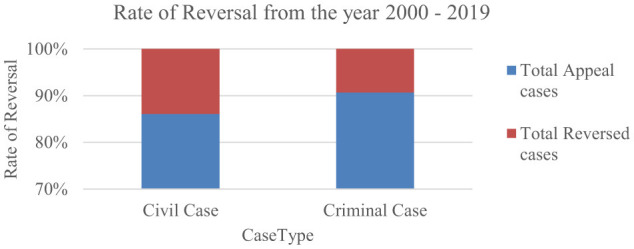
The rate of reversal in civil and criminal cases since 2000.

The appeal case will be accepted by the Supreme Court only if there is an error in constitutional law. Thus, out of 8k appeal cases, the decision was changed only for 928 cases. Figure 1 shows the rate of reversal in each of the case types among the total number of appeal cases filed in the Supreme Court.

## 5 Proposed Legal Logit Model

The suggested LLM is developed by augmenting the Multinomial Logit (MNL) model with neural network elements. The reason for this selection is 2-fold. Initially, MNL is extensively applied in discrete choice analysis because it has a robust theoretical basis and interpretability. MNL allows for estimating parameters that simply illustrate how certain factors, i.e., case facts, lower court decisions, or judge-specific attributes, affect judicial decisions. But the classical MNL is limited by its linearity and Independence of Irrelevant Alternatives (IIA) assumptions, which constrain it from detecting intricate high-dimensional interactions in judicial judgments. In contrast, neural network models like DNNs are capable of detecting such implicit non-linearities but tend to be black-box predictors that fail to give transparent reasons for the variables behind the decision. Likewise, MNL latent-class extensions (L-MNL) can account for heterogeneity but are still unable to learn complex feature interactions in a flexible manner. The LLM avoids these shortcomings by integrating the transparency of MNL with the expressiveness of neural networks. In the design, the neural layers learn non-linear relationships between features, while the MNL head maintains the discrete choice framework and makes the impact of various factors transparent. Its hybrid structure also renders LLM highly appropriate for the legal field, where interpretability as well as predictive accuracy are imperative. The experimental evidence in this study substantiates that LLM performs better than standard logit and neural baselines, confirming its efficacy in predicting judicial disagreement. Hence, this section begins with the introduction of the discrete choice model and an elaborate explanation of the MNL model, followed by its application in a neural network framework, with a greater emphasis on feature selection.

### 5.1 Discrete Choice Model

According to the survey, Discrete Choice Models (DCM) operate within the framework of typical decision-making. It is anticipated that when presented with a discrete set of options, people select the one that offers the most significant benefit or utility. This suspicion implies that the utility of a decision is a function of the quality of the alternatives and the characteristics of the person making a choice. DCM allows regular people to take into account factual knowledge about the practical bounds. By fitting a DCM to a dataset of transportation mode decisions, experts noticed that the travel mode choice is related to both qualities of the decision and to collaborations between the decisions and characteristics of the decision maker. The MNL is the accepted practice in DCM. Due to its appealing characteristics in terms of a thorough embedding in economic theory, it is frequently the starting point for modeling choice decisions.

One type of decision-making framework is the DCM, in which the decision-maker selects one option from several possible outcomes (Michael and Nagler, 1998). A Discrete Choice Experiment (DCE) is a quantitative method for suggesting individual preferences. It enables scientists to understand how people value their chosen attributes by asking them to express their preferences over various theoretical alternatives. In DCE, the set of options, also called the choice set, must have the following three properties to work within a discrete choice framework:

˙ Choices must be mutually exclusive.
˙ Options need to be fully explored.
˙ There should be a finite number of choices.

#### 5.1.1 Discrete choice framework

Travel mode choice research was one of the earliest uses of the discrete choice framework created in the 1970s by McFadden (1973) and others working in the field of travel demand (i.e., the choice between train, bus, car, or airplane). A later application of the approach involved picking out travel itineraries and vacation spots (Ben-Akiva and Lerman, 1985). This research uses the DCE framework to analyze a judge's preference for a case. Four assumptions make up the discrete choice framework (Ben-Akiva and Bierlaire, 1999):

- *Authorized to make choices*: Someone or something that makes a decision is called a decision maker.
- *Alternatives:* From the collection of all feasible options (alternatives), a decision maker must select one option.
- *Attributes:* Attributes of alternatives that appeal to the decision maker are discussed. A decision maker weighs the merits of each possible course of action. The decision maker itself may have a set of characteristics.
- *Guidelines for making a choice:* Following the commands of economic theory, a person making a choice will pick the option that maximizes their (anticipated) utility (net gain, profits, and satisfaction).

The i-th decision maker faces a set of j possible courses of action. To clarify, alternatives refer to the available options for selection, while “choice” refers to the final decision made by the decision maker. The term “option set” is sometimes used to refer to all potential solutions; however, “set of alternatives” may be more accurate.

#### 5.1.2 Multinomial Logit Model

The MNL model is a variant of the Discrete Choice Model used for calculating the probabilities of selecting various alternatives. The model is attractive because it is stochastic, yet it allows for consideration of various decision factors. It has been used as a promotional tool by several authors. A variant of this model is used to illustrate the likelihood that a car buyer purchases a vehicle from a specific dealership by Hlavac and Little (1966). The model is integrated into the pre-test market evaluation procedure for new products developed by Silk and Urban (1978). The logit is used by Punj and Staelin (1978) to characterize how potential business school students make their decisions. The broad explanation and comparison of the logit's fitting ability to that of regression for shoppers' choice of grocery stores are provided by Gensch and Recker (1979). The logit has an even more extensive history of use in transportation planning, particularly in determining the best way to anticipate an individual's preference for a given mode of transportation (e.g., vehicle vs. bus) (Domencich and McFadden, 1975).

The MNL model assumes that the decision-maker chooses an alternative that gives them maximum utility (Utility can be measured in terms of an individual's satisfaction, benefit, usefulness, etc., depending on the individual). The utility is coded as *U*_*i, j*_ if a decision maker (judge) i ∈ I obtains a maximum utility by choosing an alternative (factor) from j ∈ J choices. The utility is given as follows if the decision-maker chooses $\hat{ȷ}$


(1) $$\begin{array}{l} U_{i,\hat{ȷ}}>U_{i,j};\forall j\neq \hat{ȷ} \end{array}$$

In general, the utility function is unobservable. For each of the alternatives given to the user or decision maker, some features are associated, denoted as *x*_*i, j*_∀ *j*. Not only are the alternatives associated with the features, but the decision maker itself also possesses certain features. So, the alternatives that are chosen are dependent on the decision maker's characteristics. The features of the decision-maker are represented as *s*_*i*_. The unknown decision-maker's utility and observed features are related as


(2) $$\begin{array}{l} V_{i,j}=V\left(x_{i,j},\;s_{i}\right) \end{array}$$

Where *V*_*i, j*_ is considered to be a linear combination of features. Given an individual i and a set of d variables. For convenience, *V*_*i, j*_ in Equation 2 is represented as


(3) $$\begin{array}{l} V_{i,j}=\sum_{d}\beta_{d}.\;x_{dij} \end{array}$$

Where β are preference parameters associated with input features. For example, if a court is trying to predict the case, then the simple model is represented as


(4) $$\begin{array}{l} V_{i,j}=a^{*}case_{i,j}+b^{*}Judge_{i} \end{array}$$

where a and b are learnable parameters by the model. The relationship between *V*_*i, j*_ and *U*_*i, j*_ is denoted as


(5) $$\begin{array}{l} U_{i,j}=V_{i,j}+\;E_{i,j} \end{array}$$

$E_{i,j}$ is unobservable factors that affect the utility but are not considered in *V*_*i, j*_. The probability of choosing an alternative $\hat{ȷ}$, should give maximum utility rather than choosing the alternative j. This is represented as


(6) $$\begin{array}{l} P_{i,j}=P\left(U_{i,\hat{j}}>U_{i,j};\forall j\neq \hat{ȷ}\right) \end{array}$$

$E_{i,j}$ is said to be i.i.d (independent and identically distributed), the MNL has a property


(7) $$\begin{array}{l} P_{i,j}=\frac{exp\left(V_{i,j}\right)}{\sum_{k=1}^{j}exp\left(V_{i,j}\right)} \end{array}$$

Finally, the model is optimized as


(8) $$\begin{array}{l} \hat{\theta }=\text{arg max}\sum_{i\;\in \;I\;}\sum_{j\;\in \;J\;}y_{i,j}\ln\;\left(P_{i,j}\right) \end{array}$$

Where *y*_*i, j*_ is an indicator of whether decision maker i is associated with the alternative j.

### 5.2 Proposed Legal Logit Model

DCMs are typically developed in a random utility model, assuming decision-makers aim to maximize their utility (Long, 2001). Rational choice theory has given rise to the preeminent paradigm of judicial decision-making, in which the judge is a rational actor who applies the law, precedent, and reasoning to arrive at a verdict (Posner, 1998). However, it is well-known that this model can account for just a fraction of the process. Various scholars have used this model, transitioning (Fisher and Reed, 1998) from the legal realists of the early half of the twentieth century to the critical legal theorists of today.

It is not enough to look at the facts when deciding how to proceed with a case. Therefore, the court's decision considers the facts of this case, the outcomes of similar instances, and the judge's qualifications and experience. As the choice model (Section 5) suggests, individuals select solutions that best fit their needs and circumstances. Predicting the factors that ultimately decide a case is the goal of the LLM—Legal Logit Model. The MNL, a notable example of DCM, can foretell what factor or factors influenced a Supreme Court justice in a given case. The proposed model aims to identify the predictive factors that influence a judge's decision in a case. Therefore, greater emphasis is given on feature engineering, ensuring that the addition of numerous important features to the model does not lead to dimensionality issues.

#### 5.2.1 Selecting important features

To extract the desired information from unstructured data, it is first transformed into structured data with the required attributes. The proposed model uses Named Entity Recognition (NER) implemented in the Python NLTK library. Table 1 shows the extracted features. Considering the features such as judge-specific and case-specific (Sivaranjani and Jayabharathy, 2023), as well as lower court features given in Katz (2013), the proposed model incorporates additional features, including salience and social impact, which are categorized as other factors. Including additional features, the proposed model gives better prediction accuracy and the most predictive factors in the case. The features are categorized as follows:

▪ Judge Specific Features
▪ Historic Judge Features
▪ Case-Specific Characteristics
  ▪ Facts
  ▪ Law
▪ Lower court features
▪ Other Factors
  ▪ Salience
  ▪ Social Impact

**Table 1:** Features extracted by the feature engineering mechanism.

**Features**
**Judge specific feature (mean values)**
• Justice direction
• Justice direction petitioner
• Justice direction respondent
• Justice direction for circuit origin
• Justice direction for circuit source
• Justice direction by facts
**Case-specific feature**
• Case origin
• Case origin circuit
• Source information
• Source circuit
• Law type
• LC decision direction
• LC decision
• LC dissent
• Case facts
• Case place
• Argument month
• Decision month
• Petitioner
• Petitioner bin
• Respondent
• Respondent bin
• Certiorari
**Lower court features (mean values)**
• LC direction source circuit
• LC direction facts
• LC direction petitioner
• LC direction respondent
**Other factors (1—Yes, 0—No)**
• Salience
• Social impact

##### 5.2.1.1 Judge specific features

When a case is to be judged, judgment does not depend solely on the case-specific features. As the choice model represents, each person chooses different options based on their experience and those that are most suitable for the current situation. Determining a choice involves the characteristics of an individual. Similarly, forecasting judgment on a specific case should also consider features of the judge, such as the party that appointed the president, the judge's direction, salience, etc., to improve the performance. Table 1 shows the features considered for each judge.

##### 5.2.1.2 Historic judge features

During the feature engineering, along with judge-specific features, the essential parameters that a judge possesses concerning the court are also included. This is used to calculate baseline trends in court and judge behavior. These features show how conservative or liberal a judge is in giving the judgment. This feature is calculated by taking the mean value of considering the number of cases a particular judge handled and the number of cases the judgment was reversed throughout their career. Conservative is coded as 1, and liberal is coded as 0.

##### 5.2.1.3 Case-specific features

The model takes case-specific information such as the issue, issue Area, law Type, cert, Reason, respondent, petitioner, case Origin, case source, and LC Disposition Direction, which are predictive factors for a judge to make a confident decision. Given the petitioner and respondent features, the model bins the features into similar groups in higher-order bins, which may increase prediction accuracy.

#### 5.2.2 Implementing Multinomial Logit in convolutional neural network

**Figure 2:**
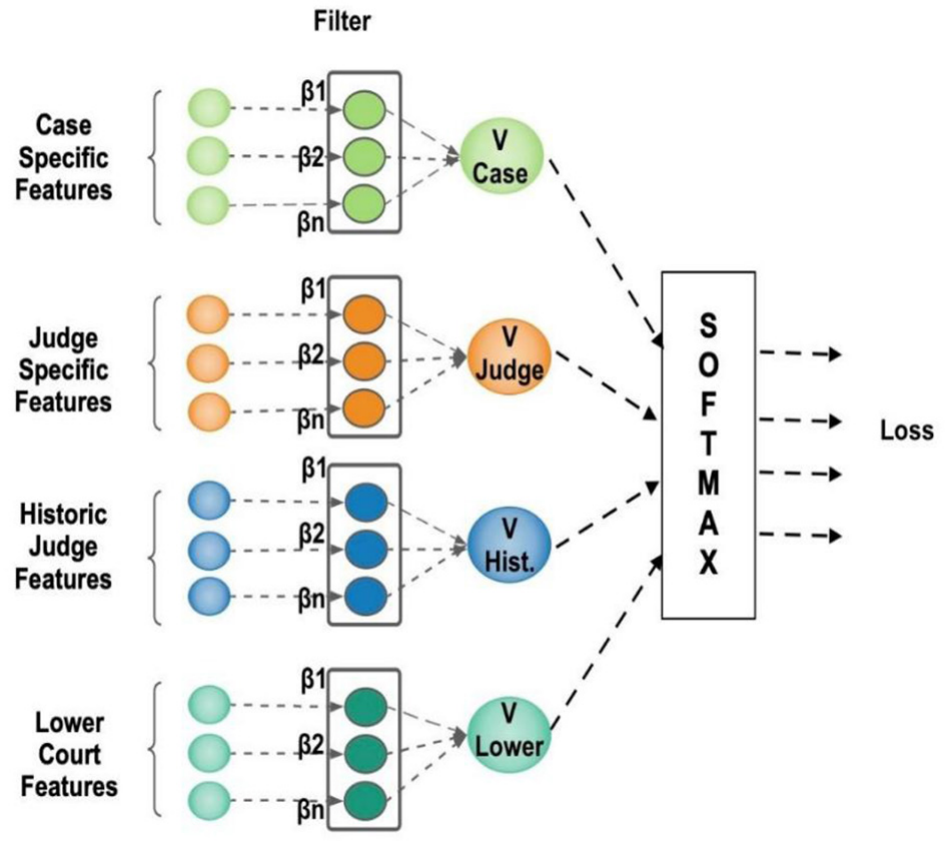
Architecture of proposed Legal Logit Model.

Figure 2 depicts the architecture of the proposed LLM using a Convolutional Neural Network with two hidden layers working like an MNL model. The case-specific input features are divided into four alternatives: Facts, Law, lower court decisions, and other factors that may influence the decision maker. *Case Specific Features*—These are characteristics (facts of the case and law involved) that define the case at issue. They come as a collection of inputs (green circles) located on the left-hand side, with the names “Case Specific Features” and *Judge Specific Features*. These are the characteristics that define information specific to the judge in this instance. They are drawn as orange circles and labeled as “Judge Specific Features.” *Historic Judge Traits—*These are historical information or previous actions regarding the judge (blue circles). *Lower Court Features—*These are the traits or facts of the lower court involved (teal circles).

Every feature group is one input vector with a quantity of values (designated by a quantity of circles). The LLM model is applied to the i × d matrix, where i refers to the decision maker, and d refers to variables that vary across i. The data set used to estimate the LLM model has a data format comparable to that of most other regression models, as well as numerous event spreadsheets. Each column denotes a variable (a characteristic of the alternative), and each row (a record) denotes an observational unit (a decision maker). The alternative chosen from a list of alternatives is indicated by the dependent variable, which is a nominal variable. The variation in the characteristics of decision-makers determines the variation in outcomes. The Neural Network takes all the features as input, along with the weight and bias. Since decision-makers' features cannot be directly fed into the Neural Network, the vector of decision-maker characteristics is embedded with the choice vector. This is the dot product of decision-maker features and case features, resulting in a vector. The embedding converts categorical attributes to low-dimensional real-valued vectors. Each of the attributes v ∈ |V| is converted to *R*^E*1^, $W_{embed}\;\in R^{V*E}$, where V represents the size of the attributes, and E represents the embedding dimension. This reduces the size of the input matrix dimension and is efficient in terms of computation.

Once the embedded vector of all the features is given as input, 1st hidden layer of the CNN imitates the MNL algorithm to calculate the utility value by an adaptive linear transformation, then Equation 5 is given as


(9) $$\begin{array}{l} U_{k}=\left(X_{k}^{*},\;\beta_{k}^{\left(1\right)}\right)+b_{k}^{\left(1\right)},k\in \text{J} \end{array}$$

Where J denotes all the alternatives e.g., J = {Facts, Law, lower court decision, other factors}. $X_{k}^{*}\in R^{d_{k}}$ is the input to the Neural Network of the kth category of alternative. $W_{k}^{\left(1\right)}\;\in R^{d_{k}}$ and $b_{k}^{\left(1\right)}$ are the weight and bias of the first hidden layer.

The 2nd hidden layer receives the input as the utility from the 1st hidden layer. The 2nd hidden layer enhances feature representation through fully connected neurons. It then calculates the relevance of all the utility values received from the previous layer. Finally, the softmax layer calculates the choice probabilities between 0 and 1. The hidden layer calculations are given as follows:


(10) $$\begin{array}{l} H^{\left(1\right)}=\left[\;U_{facts},\;U_{Law},U_{Lower\;Court\;Decision},U_{other\;factors}\right] \end{array}$$

In CNN, the weight matrix is in the shape of a filter used to connect the hidden layer *H*^(1)^ to the next hidden layer by applying convolution operations. Hence, the value of the i-th neuron in the layer (*H*^(2)^) is given as


(11) $$\begin{array}{l} H^{\left(2\right)}=g\left(\sum_{k=0}^{d}h_{\left(t.i+k\right)}^{\left(1\right)}\beta_{k}^{\left(2\right)}\right)+b_{i}^{\left(2\right)} \end{array}$$

Where {β_1._, ……..β_*d*._} = β is (1 × d) filter, t is the stride, and *b* is bais and the activation function is represented as *g*.

As represented in Equation 3, the utility function Vi = {*v*_1*i*._, ……..*v*_1*i*_} is calculated by retrieving the MNL formula in a single layer (*H*^(1)^), activity function as (f(x) = x) and stride t to d.

Then the softmax activation function gives the probability calculated as


(12) $$\begin{array}{l} \left(\sigma \left(V_{i\;}\right)\right)j=\;\frac{exp\left(V_{i,j}\right)}{\sum_{k=1}^{j}exp\left(V_{i,j}\right)} \end{array}$$

This is identified as the overall probability as defined in Equation 8. Then the output of the softmax function is given to the categorical cross-entropy loss function.


(13) $$\begin{array}{l} H_{i\;}\left(\sigma ,\;y_{i}\right)=-\sum_{i\;\in \;I\;}\sum_{j\;\in c_{n}}y_{i,j}\log\left(\sigma \left(V_{i\;}\right)\right) \end{array}$$

Where *y*_*i, j*_ is an indicator of whether decision maker i is associated with the alternative j.

## 6 Experimental setup and evaluation

The dataset was divided randomly into 70% training, 10% validation, and 20% testing. The model parameters were estimated with the training set, hyperparameters were tuned to avoid overfitting with the validation set, and generalization performance was measured with the test set. All Neural Network-based models depend heavily on hyperparameters. Table 2 summarizes the values of each hyperparameter on which the model seems to perform well. ReLu and softmax use two activation functions in the hidden and final layers, respectively. The cross-entropy loss function is combined with L1 and L2 penalties to calculate the actual and predicted values error. The number of hidden layers is defined to be 8 with 100 neurons. Increasing the hidden layer by more than 8 does not affect the output accuracy. To protect the model from overfitting, the dropout rate is defined as 0.2 and the learning rate as 0.001. The optimal number of iterations for the proposed model is 500, as a few iterations lead to underfitting the model, and a larger number of iterations leads to overfitting. The proposed and existing neural (DNN, NN-MNL, and L-MNL) models use the Adam optimizer and have run for 100 epochs. Each model's parameters are initialized using the Keras Python Deep Learning Library (Chollet, 2015) default random initialization. It is observed that there is no difference in learning time among models.

**Table 2:** Hyperparameter tuning for LLM model.

**Hyperparameter**	**Values**
Activation function	ReLu in hidden layers and softmax in the final layer
Loss function	Cross–Entropy
Number of layers (L)	8
Number of neurons (H)	100
L1 loss	1.0, 0.5
L2 loss	1.0, 0.5
Dropout	0.2
Batch normalization	True, false
Learning rate	0.0001
Number of iterations	500
Mini batch size	250

### 6.1 Experimental results and discussion

This section shows the performance comparison of existing models with the proposed model. The model is evaluated using two performance metrics, namely accuracy and Monte Carlo estimation, to assess its performance. Monte Carlo is used to evaluate the outcome of an uncertain event. The training set consists of 170 judges' features and case-specific features. The results are evaluated by repeating the experiment 100 times.

#### 6.1.1 Accuracy

**Figure 3:**
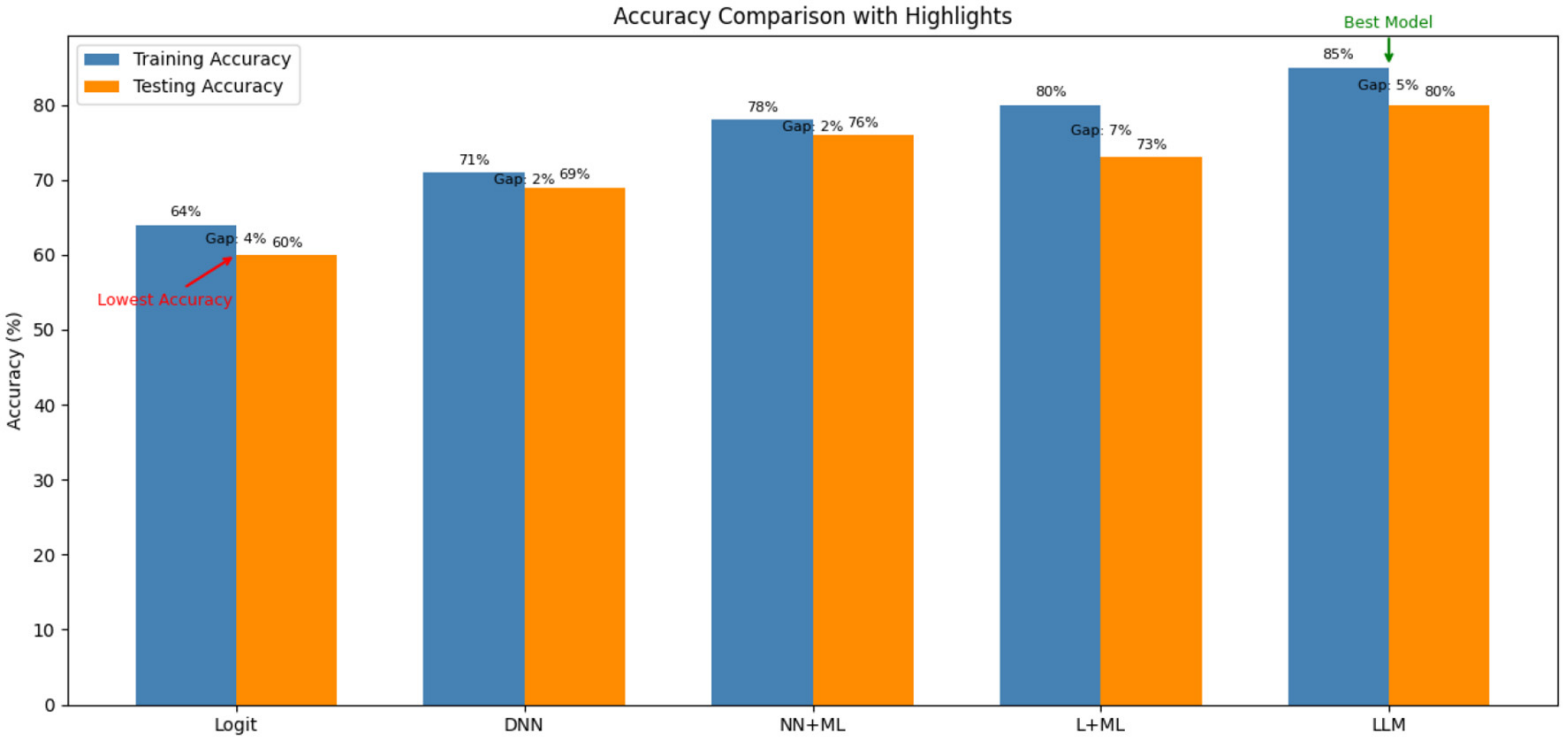
Accuracy comparison of the proposed model with existing models.

Figure 3 shows the accuracy of the proposed and existing models. The proposed model is compared with traditional MNL and Neural Network-based MNL models: DNN, NN-MNL, and L-MNL. The relative error terms *e*_β_ and *e*_β_*i*_/β_*j*__ must be defined to calculate the model's accuracy, where β is the preference parameter. The error terms are defined as


(14) $$\begin{array}{l} e_{\beta }=\;|\frac{\beta -\;\hat{\beta }}{\beta }| \end{array}$$

(15) $$\begin{array}{l} e_{\beta_{i}/\beta_{j}}=\;|\frac{e_{\beta_{i}}-e_{\beta_{j}}}{1-\;e_{\beta_{j}}}| \end{array}$$

The value clearly shows that the proposed LLM model outperforms the existing models. The proposed model improves the parameter estimation by reducing the error rate. From the graph, it is observed that the MNL model still performs equally to the Neural Model despite having a high relative error in parameter estimation. Other Neural Network models have errors in parameter estimation. The NN component in the proposed model corrects the estimators by learning simply from the information included in the linear component's data. Both the MNL and the LLM models are effective at retrieving the parameter ratio.

#### 6.1.2 Monte Carlo average log-likelihood estimation

The broad category of computational calculations known as Monte Carlo procedures or Monte Carlo tests uses repeated random sampling to produce numerical results. The underlying concept is to use randomness to address problems that may initially be deterministic. They are frequently used in mathematical and physical problems and are particularly useful when applying other strategies would be difficult or unthinkable. To ensure that results were not dependent upon a single individual random partitioning, a Monte Carlo simulation was performed by running the random sampling process multiple times. Then the metrics of performance were averaged across these iterations. This approach reduces variance due to sampling noise and provides a more robust estimate of model accuracy, making the evaluation less sensitive to any specific train–test split.

Log Likelihood estimation is a degree of the goodness of fit for any model. The higher the value, the better the model. The value of Log Likelihood can range between –∞ to +∞. The model log Likelihood estimation for independent and identically distributed data is given as follows:


(16) $$\begin{array}{l} ln\;p\left(f|\theta \right)=\;\sum_{i=1}^{N}ln\;p\left(f_{i}|\theta \right) \end{array}$$

Where f is the discrete random variable, which ranges from 1 to n, and p is the probability, θ is the parameter distribution. *L* is the likelihood function.

Using Monte Carlo's method, random samples are generated from the input data, and the log-likelihood is calculated for the model. This is repeated 100 times, and the average likelihood value is calculated to find the best fit of the model.

**Figure 4:**
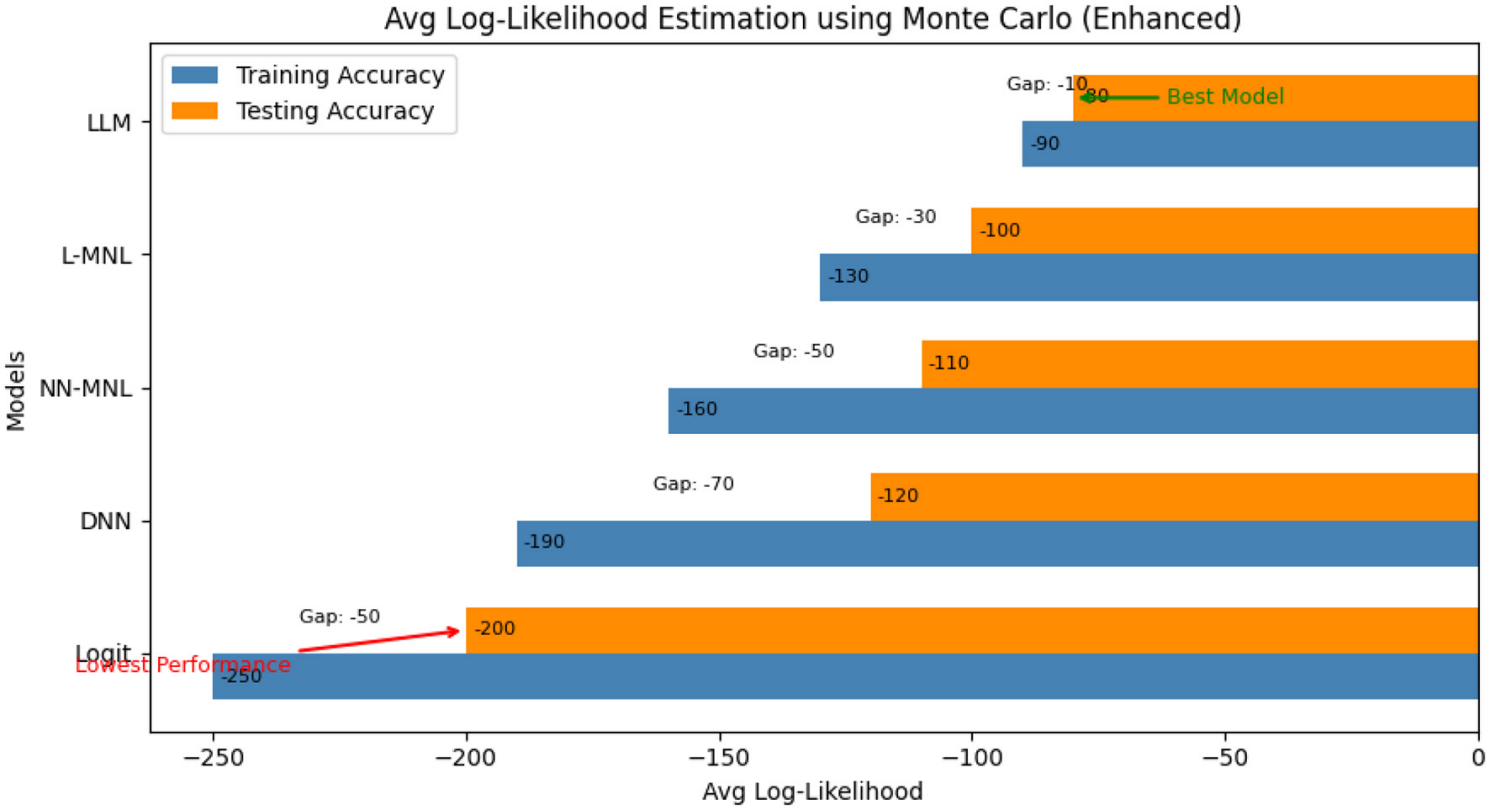
Average log-likelihood estimation using Monte Carlo.

Figure 4 shows the value of the Average Log-Likelihood estimated using Monte Carlo. The graph illustrates that the neural network-based choice model provides a better fit to the data than the traditional MNL model. Moreover, we observed that the proposed model has a lower likelihood value than the existing neural model.

The LLM performance is uniformly strong according to all the measures of evaluation. Its log-likelihood estimates (Training = −90, Testing = −80) support both a fit and low overfitting, as evidenced by the low gap (−10). The 80% accuracy further confirms that the model accurately captures case outcomes. Apart from accuracy, the AUC of 0.78 also validates that the model has excellent discriminative ability in separating different alternative outcomes. Furthermore, the F1-score of 0.76 offers balanced recall and precision to ensure robustness even in class-imbalanced instances. Together, these metrics indicate that the LLM not only fits the data well but also generalizes effectively to provide a complete and dependable predictive model.

#### 6.1.3 Training and inference complexity

Although accuracy and log-likelihood are significant indicators of predictive accuracy, efficiency in terms of training and inference complexity of the model is also vital, particularly for large judicial datasets. Table 3 compares the computational complexity of conventional and neural network–based discrete choice models. The comparison in this analysis highlights the scalability trade-offs between understandable models, such as MNL, and more expressive but computationally expensive models like NN-MNL and LLM.

**Table 3:** Asymptotic training and inference costs of models.

**Model**	**Training complexity (per epoch/iteration)**	**Inference complexity (per case)**	**Justification**
Logit/MNL	*O*(*N*.*k*.*d*)	*O*(*k*.*d*)	Linear in #cases (*N*), #alternatives (*k*), and feature dimension (*d*).
L-MNL (latent classes/random coeffs.)	*O*(*N*.*k*.*d* + *N*.*G*.*d*) (per EM/MC step)	*O*(*k*.*d*)	Additional overhead from G latent classes; inference is the same as MNL.
DNN (softmax classifier)	*O*(*N*.*P*), *where P* = ∑*h*_*l*_*h*_*l*+1_	*O*(*P*)	Complexity scales with parameter count (*P*); final softmax adds k.
NN-MNL (NN feature extractor + MNL head)	*O*(*N*.*P* + *N*.*k*.*d*_*out*_)	*O*(*P* + *k*.*d*_*out*_)	Neural network extracts a representation of size *d*_*out*_, then the MNL head computes choice probabilities.
LLM-based (proposed Legal Logit Model)	*O*(*N*.*L*.*S*.*H*^2^)	*O*(*L*.*S*.*H*^2^)	CNN-based hybrid of MNL and NN; cost grows with the number of CNN layers (*L*), sequence length (*S*), and hidden neurons per layer (*H*).

All the variables follow the same definitions as introduced in the text.

As presented in Table 3, the traditional MNL is computationally the simplest, which varies linearly with cases (N), alternatives (k), and feature dimension (d). L-MNL adds training cost because of latent classes (G), while inference is the same as MNL. DNN and NN-MNL add neural network feature learning, whose complexity depends on the number of parameters (P) and the output representation size *d*_*out*_. The proposed LLM model is realized through a Convolutional Neural Network (CNN) design where feature embeddings are fed through several convolutional layers before the use of an MNL-similar softmax choice head. This adds complexity in training, growing with the number of layers (L), sequence length (S), and hidden neurons per layer (H). Despite this added expense, the LLM is the top predictor (see Table 4). Thus, it is best applied to legal prediction problems where the simulation of non-linear interactions between case-specific and judge-specific features is important.

**Table 4:** Comparative performance of models (log-likelihood and predictive metrics).

**Model**	**Test log-likelihood**	**Accuracy (%)**	**AUC**	**F1-score**
Logit	−200	66.0	0.62	0.60
DNN	−120	70.2	0.67	0.65
NN-MNL	−110	73.5	0.70	0.68
L-MNL	−100	76.8	0.74	0.72
LLM	−80	80.0	0.78	0.76

#### 6.1.4 The parameter/features influencing decision-making

The above results show the performance of LLM and prove that it outperforms the other existing algorithms. However, the results do not indicate which feature or parameter led the judge to choose the alternative that yields the highest utility. Most of the choice models try to improve the predictability of the alternative; only a few concentrate on predicting the influencing features that aid in choosing the alternative. The legal practitioner highlights the aspects and questions in each case and places great importance on legal belief and its contribution to jurisprudence. Although many studies on legal research by legal scholars and social scientists examine the behavior of the Supreme Court, the challenging part lies in portraying the social factor and understanding how the law drives the outcome. Table 5 shows the feature score calculated during the feature extraction. These scores give much information on their contribution toward legal prediction. Many highly correlated features complicate their analysis (Tolos and Lengauer, 2011; Strobl et al., 2008).

**Table 5:** Feature score to determine predictive factor.

**Features**	**Values**
**Case-specific feature**	
Case origin	0.00971
Case origin circuit	0.00845
Source information	0.00953
Source circuit	0.01015
Law type	0.0137
LC disposition direction	0.0119
LC disposition	0.01125
LC dissent	0.00706
Case facts	0.01541
Case place	0.01469
Argument month	0.02014
Decision month	0.01349
Petitioner	0.01406
Petitioner bin	0.01199
Respondent	0.0149
Respondent bin	0.01179
Certiorari	0.01408
**Lower court features (mean values)**	
LC direction circuit source	0.00962
LC direction facts	0.01334
LC direction petitioner	0.00949
LC direction respondent	0.00973
**Judge specific feature (mean values)**	
Justice direction	0.01248
Justice direction petitioner	0.00732
Justice direction respondent	0.00724
Justice direction for circuit origin	0.00792
Justice direction for circuit source	0.00891
Justice direction by issue	0.01881
**Other factors (1—Yes, 0—No)**	
Salience	1 or 0
Social impact	1 or 0

Table 4 provides an insight into the contribution of different classes of features. The contribution values indicate legal features (e.g., Case Facts = 0.01541, Argument Month = 0.02014, Justice Direction by Issue = 0.01881) to be the core of predictive performance. They comprise the factual content of a case and judicial leaning, which by nature are more predictive in significance. Conversely, social attributes (Social Impact, Salience) are binary features (1/0) of general societal salience. While their quantitative contribution is smaller than for legal attributes, they are contextual qualifiers. For example, a highly public or politically sensitive case might make non-standard judicial conduct, like dissent, more likely. Social Impact labels cases with immediate impacts on society; while its quantitative contribution is smaller than for legal attributes, its presence can make predictions fundamentally different. Broadly, the model is based on legal content (petitioner/respondent attributes, facts, judicial guidance by issue), with social context features refining predictions to suggest where judicial decisions are set to diverge from purely legal analysis. Case-specific features account for approximately 24% of the contribution, with other classes combined accounting for approximately 7%. Interestingly, in this setup, social impact-marked cases have specific influence, suggesting political and social salience heavily interact with legal reasoning in judicial decision-making.

## 7 Inferences

The results show that the proposed LLM outperforms the other models considered. The LLM improves parameter estimation while minimizing error rates. In contrast, the traditional logit model performs well only under linear data structures and fails to capture non-linear relationships. Neural models such as DNN and NN-MNL perform better than the classical logit but still suffer from errors in parameter estimation because they primarily treat inputs through linear dependencies.

Figure 3 illustrates the behavior of the proposed model when evaluated from the 0th neuron to the 5,000th neuron in a single-layer (L = 1L = 1L = 1) configuration. It is observed that the NN component does not fully capture all non-linearities between the 0th and roughly the 20th neuron, as reflected by higher likelihood values and unstable parameter estimates. Between the 20th and 100th neurons, the values stabilize and align with the ground truth. Beyond this range, the model closely approximates the true specification, indicating that the NN component has successfully learned the underlying non-linearities.

Whereas, the LLM identifies attributes strongly associated with judicial disagreement, such results should not be interpreted as evidence of causation. The model reveals predictive structures inherent in the data rather than establishing causal determinants of judicial behavior. Establishing causal relationships in observational legal data remains a distinct methodological challenge, as emphasized in the causal inference literature (Pearl, 2009; Rubin, 2005; Gelman and Imbens, 2013). Accordingly, this study contributes a predictive framework that can inform further inquiry but does not claim causal interpretation.

## 8 Conclusions

This study proposed a model that identifies the differences of opinion among lower courts and the Supreme Court, as well as the predictive factors that influence a judge's decision. With aspect-based feature extraction, the LLM learns utility by design, considering only the appeal cases from the entire Supreme Court dataset. This improves the predictability performance with a testing accuracy of 80%. The model also calculates the probability of each aspect contributing to the final decision, along with the judge's specific feature. We compared our model performance with other existing algorithms, and the results demonstrated that the proposed LLM outperforms the other neural-based as well as traditional discrete choice models in terms of predictability and accuracy. In the future, this study could be extended to improve the accuracy by identifying more hidden factors from the case and judgment.

## Data Availability

In this study, publicly available datasets were used to train and test the model after converting them into a format compatible with the model. The data can be accessed at: https://indiankanoon.org/browse/supremecourt/.

## Appendix

***Dataset Source:***

The dataset is downloaded from the Indian Supreme Court Public Database (provide precise link).

***Pre-processing Steps:***

Raw case records were downloaded and filtered for relevant fields (case origin, petitioner, respondent, legal context, etc.).
Features were transformed into case-specific, lower court, judge-specific, and social context categories.
Binary encoding was used to categorical features (e.g., Salience, Social Impact).
Train-test splits were created (80:20).

***Software Environment:***

The experiments were carried out in Python (v3.10). The major libraries employed are described below:

pandas (v1.5.3): Data manipulation and preprocessing
numpy (v1.24.2): Numerical computations
scikit-learn (v1.2.2): Feature encoding, model training, evaluation (Accuracy, AUC, F1-score)
statsmodels (v0.13.5): Discrete choice modeling (Logit, MNL, L-MNL)
tensorflow/keras (v2.12.0): Neural network models (DNN, NN-MNL)
matplotlib (v3.7.1) and seaborn (v0.12.2): Visualization and plots
